# In Vivo Evaluation of the Oral Toxicity of the Chlorobutanol

**DOI:** 10.3390/toxics10010024

**Published:** 2022-01-07

**Authors:** Dahye Jeong, Hyosook Shin, Jinhee Lee, Junyoung Yang, Kikyung Jung, Jayoung Jeong, Hansol Won, Jaeho Oh

**Affiliations:** Division of Toxicological Research, National Institute of Food and Drug Safety Evaluation, Ministry of Food and Drug Safety, 187, Osongsaengmyeong 2-ro, Cheongju 28159, Korea; jdah1017@korea.kr (D.J.); aqua978@korea.kr (H.S.); tod98@korea.kr (J.L.); yangjy@korea.kr (J.Y.); kikyung@korea.kr (K.J.); 0jjy@korea.kr (J.J.)

**Keywords:** chlorobutanol, single toxicity, repeated-dose 28-day oral toxicity, NOAEL

## Abstract

Chlorobutanol (CB) is used as a preservative in cosmetics and has antibacterial activity. This study investigated the single- and repeated-dose 28-day oral toxicity of a CB solvent in Sprague Dawley (SD) rats. For the single-dose oral toxicity study, a dose of 62.5, 125, or 250 mg per kg of body weight (mg/kg b.w.) of CB was given once orally via gavage. For the repeated-dose 28-day toxicity study, the high dose was set as 100 mg/kg b.w./day, and the middle, middle-low, and low doses were set to 50, 25, and 12.5 mg/kg b.w./day, respectively. Body weight was not significantly changed in the repeated-dose toxicity study. Relative liver and kidney weights were significantly increased in both sexes of the 100 mg/kg b.w./day treatment group. However, there were histopathological changes in liver and kidney for females and males, respectively. These data suggested that the approximate lethal dose (ALD) of CB was over 250 mg/kg b.w./day in the single-dose study, and the no adverse effect level (NOAEL) for CB was over 50 and 12.5 mg/kg b.w./day for female and male rats in the repeated-dose toxicity study.

## 1. Introduction

Chlorobutanol (CB; 1,1,1-trichloro-2-methyl-2-propanol), an alcohol-based preservative, has a broad spectrum of antimicrobial action in many consumer products [1,2]. Preservatives, such as CB, are included in ophthalmic solutions to maintain the shelf life of the product and to prevent contamination during the treatment period of the patient [3]. CB is also the active component in topical anesthetics and certain oral sedatives [4]. In addition, it has previously been marketed in some countries as a sedative, an antiseptic mouthwash, and a preservative in topical pastes and intra-veinous formulations of vitamins and many drugs [5,6].

Previous synthetic chemical investigations on CB were divided according to their chemical composition, including alcohols and phenols [7]. Alcohols and phenols are very effective antibacterial substances. Their action is bactericidal, especially in the case of acid-resistant bacilli [7]. According to Supriya et al., as combinations, benzyl alcohol–CB and CB enhanced the antimicrobial efficacy of the formulation against fungi [8]. Furthermore, CB is known to undergo hydrolysis at elevated temperatures, forming hydrochloric acid and other acidic reaction products [9]. This is likely to explain the change in pH of preparations that contain CB, which has been observed at 40 °C. As expected, this effect was even more pronounced at 80 °C, with both preparations showing a pH of 2.0 in 5 days [10].

Early studies reported that CB has cytotoxic effects on human corneal epithelial cells in vitro [11]. Additionally, 0.5% CB was shown to cause irritation in more than 50% of subjects [12]. In an in vitro study, 0.5% CB was observed to cause the retraction of human corneal cells, normal cytokinesis, cell migration, and cessation of mitosis [11]. It was also shown to induce the degeneration of human corneal cells and the formation of distinct membranous blebs [13]. Cases of death from suicidal overdose in large doses were reported [14,15].

Even though CB is widely used as a household chemical and is suspected to be toxic, there is no published information on the single and repeated 28-day toxicity of CB via the oral route in rats. Hence, the present study was designed to evaluate the safety of CB after a single and 28 consecutive daily oral administrations of it. In addition, this study aimed to acquire safety data for the application of household chemical substance-based preservatives, including information about the no adverse effect level (NOAEL) and target organs. As a result of this study, toxicity test data, including NOAEL and target organ information, can be applied to future risk assessments.

## 2. Materials and Methods

### 2.1. Test Substance and Vehicle

CB (C_4_H_7_Cl_3_O, molecular weight 177.46 g/mol, lot no. LRAB2932) was provided by Sigma-Aldrich (purity 95–100%, St. Louis, MO, USA). In the following tests, CB powders for oral administration were dissolved with corn oil to indicate dosing concentrations and to form the final product [16].

### 2.2. Test Animals

Male and female specific-pathogen-free Crl:CD Sprague Dawley (SD) rats (age 5 weeks) used in the single-dose and 28-day toxicity studies were purchased from Koatech Inc., (Pyeongtaek-si, Korea). The animals were adapted to laboratory conditions for 7 days after they arrived at the laboratory animal facility of the Nationsal Institute of Food and Drug Safety Evaluation of the Ministry of Food and Drug Safety (MFDS) (Cheongju-si, Korea). The studies were approved by the Institutional Animal Care and Use Committee (2020, approval no. MFDS-20-023). During the study period, every group of one or two animals with identical sex was housed in a polycarbonate cage. The animals were housed at a temperature of 22 ± 3 °C and relative humidity of 50 ± 15%. The animal rooms were maintained under a 12 h light–dark cycle and 10–20 air exchanges per hour.

### 2.3. Single-Dose Toxicity Study Design

The single-dose oral toxicity of CB in rats was examined based on the fixed-dose procedure described in the acute toxic class method of the Organization for Economic Cooperation and Development (OECD) guideline no. 423 [17] and the method of “Toxicity Test Standards for Drugs” from the MFDS [18]. Forty rats (age 6 weeks) were randomly divided into four groups (five females and five males each): vehicle, 62.5, 125, and 250 mg/kg b.w./day of CB. CB was orally administered via sonde into the stomach (gastric gavage) with respective doses in the treatment groups. The vehicle group received corn oil in the same volume, while the treated groups received CB. The rats were discreetly observed for any symptoms of toxicity immediately after the treatment and for a further 2 weeks. Observations for mortality, signs of illness, pain, injury, allergic responses, alterations in appearance, behavioral changes, such as ataxia and hyperactivity, general stimulation, and sedation were conducted every day. All the observations were methodically recorded. After 14 days of monitoring, all the rats were recorded for their body weights and sacrificed via CO_2_ inhalation.

### 2.4. Repeated-Dose 28-Day Oral Toxicity Design

The repeated-dose oral toxicity of CB in rats was performed based on the repeated dose 28-day oral toxicity study in rodents described in the method OECD guideline no. 407 [19] and the method of “Toxicity Test Standards for Drugs” from the MFDS [18]. The SD rats were randomly divided into vehicle control and CB groups, consisting of 5–7 females and 5–7 males in each group. The obtained approximate lethal dose (ALD) of CB was higher than 250 and 250 mg/kg b.w./day for males and females, respectively. Thus, the five groups were divided into 100, 50, 25, 12.5 (mg/kg b.w./day), and vehicle control. Rats were administered once daily via gavage with a CB suspension at four doses as above (100, 50, 25, and 12.5 mg/kg b.w./day) or a vehicle control (same volume of corn oil) for 4 weeks. The rats were closely observed daily for any signs of toxicity. The objective monitoring was undertaken to identify and create daily records of clear indications of toxicity, including alterations in the eyes, skin, and fur, as well as behaviors, body weight, and mortality, after the administration. In addition, the measurements of food and water consumption were recorded weekly. Then, the rats were sacrificed via CO_2_ inhalation.

### 2.5. Hematology and Clinical Biochemistry

After 28 days of dose administration, blood samples collected in heparin tubes were centrifuged at 3000 rpm for 15 min at 4 °C for the hematological analyses. The separated serum samples were used to measure the serum levels of white blood cell (WBC), red blood cell (RBC), platelet (PLT), neutrophil (NEUT), lymphocyte (LYM), monocyte (MONO), eosinophil (EOS), basophil (BASO), and reticulocyte (Retic) counts, as well as the hemoglobin (HGB), hematocrit (HCT), mean corpuscular volume (MCV), mean corpuscular hemoglobin (MCH), and mean corpuscular hemoglobin concentration (MCHC).

Hematological analysis was conducted with the whole blood collected in the EDTA-treated tubes. The alanine aminotransferase (ALT), aspartate aminotransferase (AST), alkaline phosphatase (ALP), gamma-glutamyl transferase (GGT), blood urea nitrogen (BUN), creatinine (CREA), total protein (TP), albumin (ALB), cholesterol (T-CHOL), glucose (GLU), triglyceride (TG), total bilirubin (T-BIL), direct bilirubin (D-BIL), lactate dehydrogenase (LDH), creatinine kinase (CK), uric acid (UA), calcium (CA), phosphorus (IP), high-density lipoprotein (HDL), and low-density lipoprotein (LDL) of the blood samples were determined.

### 2.6. Gross Necropsy

At the end of the study, the animals fasted overnight before the sacrifice. Animals were euthanized and then necropsied. All rats were macroscopically analyzed, as well as the individual organs, after the extraction. Samples of the following organs and tissues were gathered as follows and weighed wet without delay: liver, spleen, heart, kidney (both), adrenal gland, lung, brain, pituitary gland, thymus, urinary bladder, stomach, intestine, trachea, esophagus, thyroid gland, salivary gland, skin, femur, Harderian gland nerves, testis, epididymis, prostate and seminal vesicle (males), and uterus and ovary (females). Furthermore, the weight of every rat was measured. The relative organ weight, defined as the organ weight per 100 g of body weight at sacrifice was calculated.

### 2.7. Histopathology

All tissues were fixed in 10% neutral buffered formalin (#0151S, BBC Biochemical, Seattle, WA, USA) containing neutral phosphate-buffered saline, trimmed, processed, embedded in paraffin, and sliced into standard thin sections. Sections were stained with hematoxylin and eosin (H&E) according to routine histological techniques. The tissue slices on the slides were observed and photographed using an optical microscope (Leica DM 3000, Wetzlar, Germany).

### 2.8. Statistical Analysis

For the toxicity study, the results were expressed as the mean ± standard deviation. Statistical analyses for quantitative data containing body weight, food consumption, water intake, organ weight, hematology, and clinical chemistry data were performed using one-way analysis of variance (ANOVA), followed by post hoc Dunnett’s multiple comparison test using Graph Pad Prism 5.0 (Graph Pad Prism, San Diego, CA, USA). The level of significance was set at *p* < 0.05 to evaluate the vehicle control and treatment group.

## 3. Results

### 3.1. The Single-Dose Toxicity Study

In the high-dose (250 mg/kg b.w./day) group, one female rat was found dead approximately 2 days after the dose (Table 1). Post-dosing clinical signs in this animal included severe ataxia, dyspnea, and a moribund state. Clinical signs of the four surviving animals included mild ataxia only within 2 days after dosage. Body weight was significantly (*p* < 0.05) decreased at the seventh day of dosage in the female 250 mg/kg b.w./day group and recovered in 14 days (Table 1). The high-dose male group also showed a non-significant tendency for decreased body weight at 7 days. The ALD of CB was estimated to be greater than 250 mg/kg b.w./day in male and female rats.

### 3.2. The 28-Day Repeated Oral Toxicity Study

#### 3.2.1. Clinical Signs, Necropsy Findings, and Food and Water Consumption

During the CB administration period, there was no clinical sign related to toxicity in either sex. However, one female rat died on day 14 in the 100 mg/kg b.w./day CB-treated group (Table 2). From day 3 of administration, the dead animal had clinical signs, such as soft stool and emaciation. At the post-mortem autopsy, the animal did not show any gross findings.

The food and water intakes of the males in the high-dose group were slightly decreased in the first week. Food consumption was increased in the second week (Table 3 and Table 4). Food intake in the female high-dose group was increased in the second and third weeks compared with the vehicle control group. Food and water consumption did not show a dose-dependent pattern. Therefore, we concluded that these had no relevance to the chemical-induced toxic effect. In addition, the body weight demonstrated no significant change (Table 2).

#### 3.2.2. Relative Organ Weights

As seen in Table 5, the relative liver, kidney, and heart weights were significantly increased (*p* < 0.05) in the female high-dose group (100 mg/kg b.w./day). Meanwhile, the relative salivary gland weight was significantly (*p* < 0.05) decreased in the female high-dose group. The middle- (25 and 50 mg/kg b.w./day) and high-dose (100 mg/kg b.w./day) groups in males had significantly increased (*p* < 0.05) relative liver weights. Furthermore, the middle- (50 mg/kg b.w./day) and high-dose (100 mg/kg b.w./day) male groups had significantly increased (*p* < 0.05) relative left kidney weights. There was no significant difference in relative right kidney weights of the treated male rats compared with those from the vehicle control group, except for the high-dose (100 mg/kg b.w./day) group (*p* < 0.05).

#### 3.2.3. Hematological Changes

As presented in Table 6, the vast majority of the hematological parameters, namely, RBC, WBC, HGB, HCT, MCV, MCH, PLT, NEUT, LYM, MONO, EOS, BASO, and Retic counts, were not significantly different from the vehicle control group. However, the MCHC level, which should correspond to the level of MCV, was found to be decreased in the 50 and 100 mg/kg b.w./day male treated groups at *p* < 0.05. The RBC and EOS levels were found to be decreased in the 50 and 12.5 mg/kg b.w./day female treated groups, respectively.

#### 3.2.4. Biochemical Changes

The results of the biochemistry obtained after the 28-day repeated administration of CB are exhibited in Table 7. The vast majority of biochemical results of the male rats were not significantly affected by the CB treatment. However, there was a significant increase in the levels of ALP of male rats treated with 50 mg/kg b.w./day CB compared with the vehicle control group. Moreover, the levels of T-CHO of male rats in the 100 mg/kg b.w./day CB-treated group were higher than those in the vehicle control group. In contrast, the level of GLU was decreased among male rats CB-treated with 50 mg/kg b.w./day compared with the vehicle control group. Other statistically significant changes were observed in the female rat group. The levels of TP, T-CHO, HDL, and LDL were significantly increased among female rats treated with the higher dose levels of CB.

#### 3.2.5. Histopathological Examination

**Figure 1:**
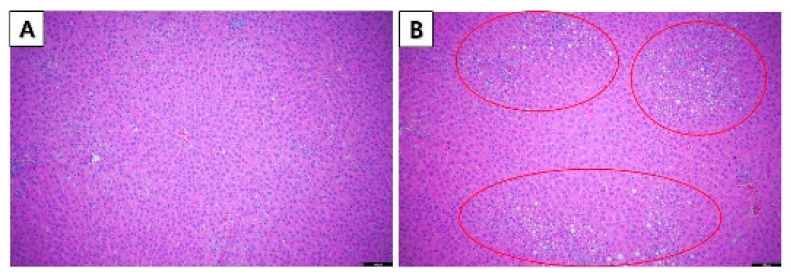
Histopathological changes in female SD rats in the 28-day oral administration toxicity test. (**A**) Normal microscopic structure of the liver of a female SD rat (×100 magnification). (**B**) Fatty change (circle) in the liver of a female SD rat administered 100 mg/kg chlorobutanol (×100 magnification). Scale bar: 100 μm.

**Figure 2:**
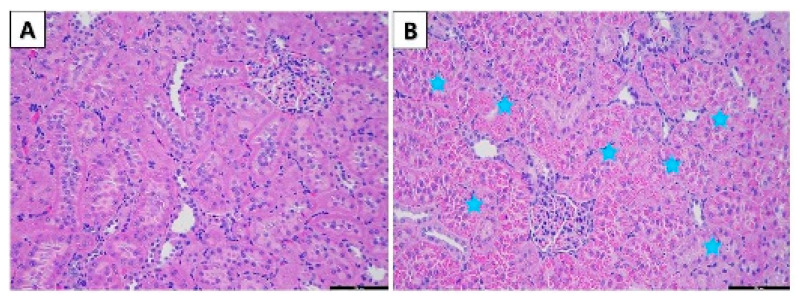
Histopathological changes of male SD rats in the 28-day oral administration toxicity test. (**A**) Normal microscopic structure of the kidney of a male SD rat (×200 magnification). (**B**) Hyaline droplet in the kidney of a male SD rat administered 100 mg/kg chlorobutanol (×200 magnification). Scale bar: 200 μm.

Histopathological observation of the stain tissues of the major organs (liver, spleen, heart, kidneys, and lungs) did not show any pathological lesions after the administration of CB at all dose levels except for 100 mg/kg b.w./day in females and 100, 50, and 25 mg/kg b.w./day in male rats. As shown in Figure 1 and Figure 2, the optical microscopy examinations of the liver and kidneys of the CB treat group and vehicle group rats are presented. Furthermore, the histopathological examination of the vehicle and CB-treated groups showed an unusual form and significant lesions in the organs. The fatty change in the liver was observed in female rats in the 100 mg/kg b.w./day group (Figure 1B). In addition, the tubular hyaline droplets were found in the kidney of male rats in 100, 50, and 25 mg/kg b.w./day in a dose-dependent manner (Figure 2B).

## 4. Discussion

Although CB is widely used as a preservative in cosmetics, sedatives, and hypnotics, there is a lack of oral toxicity assessment data. This could potentially be an obstacle for the risk assessment for cosmetics in Korea [20]. Hence, this study aimed to investigate the potential toxicity of CB.

Several previous toxicity studies reported that the CB oral LD50 in rats was close to 510 mg/kg b.w. [21]. Following these studies, we set the highest dose as 250 mg/kg b.w. in this study. Our study not only indicated that the ALD of CB was greater than 250 mg/kg b.w. for both sexes but also demonstrated the effect of a single administration of 250 mg/kg b.w. on the body weight. However, in the 28-repeated oral toxicity study, the growth rate did not show a clear adverse effect due to the CB administered at 100 mg/kg b.w./day to both male and female rats.

In our study, renal changes (increase in dose-related incidence and severity of hyaline droplets) occurred in males only and were observed at the doses of 25, 50, and 100 mg/kg b.w./day. In contrast to female rats, male rats tend to form hyaline droplets in the renal proximal tubular cells [22]. In addition, the relative kidney weight was significantly increased at 100 mg/kg b.w./day. However, there was no change in renal biomarkers, such as BUN and creatinine in serum biochemistry. In the liver, a mild increase in the fatty change was observed in females. CB is an alcohol-based preservative [7]. Based on these reports, we were interested in the side effect of CB on the liver because CB could affect the fattiness change in liver. In sum, CB produces adverse effects in female rats at 100 mg/kg b.w./day that increase the relative liver weight, as well as T-CHO, HDL, and LDL from the clinical biochemistry perspective [23,24]. Non-alcoholic fatty liver disease was shown to have an adverse effect on LDL cholesterol management using data collected from a cohort study [25].

Several studies demonstrated that chemical exposure could induce the accumulation of hyaline droplets and increase the relative kidney weight [26,27]. Renal hypertrophy, a common feature in animal models for kidney malfunction, can be caused by changes in the function of tubules [28,29]. Moreover, excessive accumulation of hyaline droplets was correlated with renal injury. Even though the BUN and serum creatinine levels are considered as the gold standard of renal injury, accompanied by histopathological findings, certain hyaline droplet nephropathies induced by chemicals do not induce serum biochemistry changes [30].

Hyaline droplets in male rats are often associated with a2u-globulin accumulation. A2u-globulin is a low-molecular-weight protein that is synthesized in the liver of male rats but is not synthesized in female rats under androgen regulation [31]. A2u-globulin synthesis is age-dependent and reached a peak at 3 months old among male rats [32]. The function of this protein is not known, but it is filtered through the glomerulus and reabsorbed by the proximal tubule under normal conditions [33]. Generally, substances that cause an increased accumulation of a2u-globulin in the renal tubules are associated with increased renal tubule epithelial degeneration, necrosis, regeneration, and subsequent renal carcinogenicity [34,35]. An enhancement of hyaline droplet formation could be predominantly caused by chemicals that induce the accumulation of α-2-microglobulin in male rat kidneys [36]. These results suggest that reduced degradation and chemical binding are associated with the renal accumulation of this protein. Accumulation of 2u-globulin is considered to be responsible for apoptosis, which stimulates cell division as a repair/regenerative response [34]. Repeated cycles of cytotoxicity and cellular replication due to the dose-dependent accumulation of hyaline droplets were thought to be associated with the renal tumorigenic response [30,37].

## 5. Conclusions

In conclusion, chemical-induced hepatocellular hypertrophy in the female liver and hyaline droplets in male kidneys were considered to be relevant to toxicity. Yet, the mechanism of toxicity was obscure, and therefore, further studies should be conducted. Based on our observational results, the NOAEL was estimated to be 12.5 mg/kg b.w./day on the basis of the renal system depression, and the lifetime safe level was estimated to be 50 mg/kg b.w./day in female rats. We expect that this study may be used for conducting risk assessments for household chemicals and improving scientific information for future studies.

## Figures and Tables

**Table 1:** The body weight and mortality of rats during the single-dose toxicity study.

			Body Weight (g)			
Sex	Dose (mg/kg)	Number Dosed	Day 0	Day 7	Day 14	Mortality (Dead/Total)
Male	0	5	176.34 ± 5.06	229.41 ± 6.94	275.50 ± 6.05	0% (0/5)
	62.5	5	176.02 ± 2.66	221.15 ± 8.16	272.97 ± 11.83	0% (0/5)
	125	5	176.49 ± 2.51	220.38 ± 4.23	274.30 ± 5.24	0% (0/5)
	250	5	176.95 ± 4.92	203.08 ± 9.15	266.49 ± 10.31	0% (0/5)
Female	0	5	131.99 ± 4.83	157.25 ± 7.37	180.22 ± 9.81	0% (0/5)
	62.5	5	131.76 ± 3.17	160.48 ± 5.99	180.22 ± 9.11	0% (0/5)
	125	5	131.66 ± 3.14	159.92 ± 9.75	182.97 ± 10.46	0% (0/5)
	250	5	131.57 ± 3.76	145.31 ± 4.56	171.35 ± 6.38	20% (1/5)

VEH, vehicle administered corn oil. Data are expressed as means ± SD (*n* = 5/group). Statistics: one-way analysis of variance (ANOVA test) followed by Dunnett’s test.

**Table 2:** Body weight changes for the 28-day repeated oral toxicity study.

			Body Weight (g)					
Sex	Dose (mg/kg)	Number Dosed	Day 0	Day 7	Day 14	Day 21	Day 28	Mortality (Dead/Total)
Male	0	5	175.5 ± 5.8	224.9 ± 8.3	269.7 ± 13.1	305.3 ± 16.4	330.6 ± 16.4	0% (0/5)
	12.5	7	175.5 ± 7.5	225.9 ± 9.7	270.1 ± 12.9	302.1 ± 13.2	328.8 ± 12.1	0% (0/5)
	25	7	175.8 ± 6.0	227.3 ± 9.1	270.7 ± 10.5	302.5 ± 17.4	325.7 ± 20.6	0% (0/7)
	50	7	175.6 ± 4.7	226.4 ± 6.4	268.2 ± 11.4	302.0 ± 13.5	327.0 ± 14.6	0% (0/7)
	100	7	176.3 ± 4.0	221.9 ± 9.2	263.1 ± 14.3	296.7 ± 24.1	324.1 ± 28.9	0% (0/7)
Female	0	5	137.1 ± 4.2	167.0 ± 4.0	185.7 ± 5.9	199.3 ± 8.0	209.9 ± 10.4	0% (0/5)
	12.5	7	137.7 ± 4.4	167.8 ± 8.5	186.5 ± 17.4	203.4 ± 21.4	210.3 ± 20.2	0% (0/5)
	25	7	138.0 ± 4.9	170.3 ± 11.8	189.0 ± 14.8	205.0 ± 17.1	213.5 ± 18.3	0% (0/7)
	50	7	137.4 ± 4.6	171.0 ± 9.3	187.2 ± 11.9	199.6 ± 12.9	210.2 ± 15.1	0% (0/7)
	100	7	138.8 ± 5.7	172.4 ± 9.9	195.8 ± 11.6	212.8 ± 7.7	202.8 ± 28.1	14% (1/7)

VEH, vehicle administered corn oil. Data are expressed as means ± SD (*n* = 5–7/group). Statistics: one-way analysis of variance (ANOVA test) followed by Dunnett’s test.

**Table 3:** Feed intake for the 28-day repeated oral toxicity study.

			Feed Intake (g)			
Sex	Dose (mg/kg)	Number Dosed	1st Week	2nd Week	3rd Week	4th Week
Male	0	5	18.3 ± 2.1	17.1 ± 2.5	18.2 ± 2.1	15.9 ± 3.1
	12.5	7	19.0 ± 0.7	17.8 ± 0.2	17.1 ± 1.6	16.4 ± 1.5
	25	7	18.2 ± 3.1	18.7 ± 1.4	17.9 ± 2.0	15.3 ± 1.6
	50	7	18.9 ± 0.8	18.7 ± 1.6	17.2 ± 1.3	16.8 ± 1.3
	100	7	14.6 ± 2.1 *	22.8 ± 4.5 *	19.8 ± 2.0	17.8 ± 3.4
Female	0	5	12.0 ± 1.0	11.4 ± 1.6	9.8 ± 1.5	10.2 ± 1.1
	12.5	7	11.7 ± 1.5	11.7 ± 1.0	11.0 ± 1.7	10.1 ± 1.3
	25	7	12.4 ± 2.1	12.2 ± 0.7	11.5 ± 1.5	12.2 ± 1.5 *
	50	7	13.1 ± 0.8	11.6 ± 1.1	10.8 ± 0.4	13.6 ± 0.4 *
	100	7	10.4 ± 1.7	12.8 ± 0.9 *	12.0 ± 2.2 *	8.6 ± 2.0

VEH, vehicle administered corn oil. Data are expressed as means ± SD (*n* = 5–7/group). * Significantly different from the vehicle control group (*p* < 0.05).

**Table 4:** Water consumption for the 28-day repeated oral toxicity study.

			Water Consumption (g)			
Sex	Dose (mg/kg)	Number Dosed	1st Week	2nd Week	3rd Week	4th Week
Male	0	5	33.3 ± 4.9	33.9 ± 3.3	36.0 ± 4.9	30.3 ± 5.7
	12.5	7	33.2 ± 1.9	32.7 ± 3.7	36.6 ± 6.2	33.2 ± 5.2
	25	7	36.9 ± 5.2	36.9 ± 5.0	36.4 ± 5.1	34.8 ± 6.8
	50	7	34.6 ± 6.6	34.5 ± 5.2	32.9 ± 6.8	31.0 ± 4.6
	100	7	23.3 ± 4.6 *	37.4 ± 6.3	30.6 ± 2.6	29.9 ± 4.4
Female	0	5	23.8 ± 1.8	23.5 ± 3.4	25.9 ± 1.6	24.3 ± 1.7
	12.5	7	24.5 ± 1.1	22.4 ± 2.5	24.3 ± 2.8	24.4 ± 5.6
	25	7	26.1 ± 2.3	22.4 ± 4.1	25.1 ± 8.4	25.6 ± 6.9
	50	7	25.0 ± 1.6	23.2 ± 4.8	23.8 ± 2.1	23.7 ± 1.9
	100	7	28.6 ± 8.2	24.6 ± 3.0	23.1 ± 2.9	15.2 ± 5.6 *

VEH, vehicle administered corn oil. Data are expressed as means ± SD (*n* = 5–7/group). * Significantly different from the vehicle control group (*p* < 0.05).

**Table 5:** Relative organ weights after the 28-day repeated oral toxicity study.

Organ (g/100 g Body Weight)	Groups (mg/kg/day)				
	0	12.5	25	50	100
Male					
Liver	2.78 ± 0.07	2.86 ± 0.14	3.00 ± 0.13 *	3.09 ± 0.09 *	3.51 ± 0.15 *
Kidney-R	0.34 ± 0.01	0.37 ± 0.09	0.35 ± 0.02	0.38 ± 0.01	0.44 ± 0.04 *
Kidney-L	0.33 ± 0.01	0.34 ± 0.02	0.35 ± 0.03	0.39 ± 0.02 *	0.43 ± 0.04 *
Adrenal gland-R (mg)	7.73 ± 0.19	7.93 ± 0.90	7.91 ± 1.04	7.68 ± 0.77	8.64 ± 1.41
Adrenal gland-L (mg)	8.00 ± 0.98	8.06 ± 1.05	7.83 ± 0.85	7.68 ± 0.73	8.08 ± 1.04
Heart	0.40 ± 0.02	0.39 ± 0.04	0.39 ± 0.02	0.40 ± 0.02	0.43 ± 0.02
Lung	0.47 ± 0.04	0.45 ± 0.02	0.48 ± 0.03	0.46 ± 0.04	0.46 ± 0.03
Brain	0.57 ± 0.03	0.57 ± 0.02	0.57 ± 0.03	0.57 ± 0.03	0.56 ± 0.05
Pituitary gland	3.16 ± 0.20	3.45 ± 0.50	3.16 ± 0.25	3.55 ± 0.30	3.27 ± 0.39
Spleen	0.20 ± 0.02	0.22 ± 0.01	0.22 ± 0.02	0.23 ± 0.02 *	0.22 ± 0.02
Thymus	0.14 ± 0.01	0.15 ± 0.02	0.16 ± 0.01	0.15 ± 0.01	0.14 ± 0.02
Testis-R	0.60 ± 0.04	0.59 ± 0.03	0.57 ± 0.03	0.57 ± 0.05	0.57 ± 0.05
Testis-L	0.59 ± 0.05	0.60 ± 0.03	0.57 ± 0.03	0.56 ± 0.05	0.56 ± 0.05
Epididymides-R	0.15 ± 0.01	0.15 ± 0.01	0.15 ± 0.01	0.14 ± 0.01	0.15 ± 0.01
Epididymides-L	0.15 ± 0.01	0.15 ± 0.01	0.15 ± 0.01	0.14 ± 0.01	0.15 ± 0.02
Prostate	0.11 ± 0.02	0.11 ± 0.01	0.11 ± 0.03	0.10 ± 0.01	0.10 ± 0.02
Seminal vesicle	0.45 ± 0.07	0.45 ± 0.04	0.43 ± 0.07	0.42 ± 0.05	0.42 ± 0.04
Salivary gland	0.19 ± 0.02	0.19 ± 0.01	0.19 ± 0.01	0.19 ± 0.03	0.16 ± 0.01
Thyroid gland-R (mg)	3.02 ± 0.62	3.10 ± 0.61	2.61 ± 0.60	2.99 ± 0.37	2.78 ± 0.29
Thyroid gland-L (mg)	3.30 ± 1.18	2.94 ± 0.30	2.67 ± 0.42	2.64 ± 0.70	2.84 ± 0.59
Female					
Liver	2.82 ± 0.15	2.88 ± 0.21	2.93 ± 0.17	2.94 ± 0.27	4.49 ± 0.99 *
Kidney-R	0.34 ± 0.02	0.35 ± 0.02	0.36 ± 0.03	0.37 ± 0.02	0.43 ± 0.10 *
Kidney-L	0.34 ± 0.02	0.36 ± 0.03	0.34 ± 0.02	0.36 ± 0.02	0.44 ± 0.12 *
Adrenal gland-R (mg)	14.48 ± 1.44	15.26 ± 2.51	15.50 ± 1.62	16.42 ± 2.64	18.35 ± 7.76
Adrenal gland-L (mg)	15.07 ± 1.97	15.07 ± 2.22	14.70 ± 2.52	16.04 ± 1.67	17.39 ± 7.25
Heart	0.42 ± 0.01	0.42 ± 0.03	0.41 ± 0.02	0.44 ± 0.03	0.50 ± 0.04 *
Lung	0.55 ± 0.02	0.57 ± 0.03	0.56 ± 0.04	0.56 ± 0.04	0.58 ± 0.06
Brain	0.82 ± 0.07	0.86 ± 0.08	0.81 ± 0.07	0.82 ± 0.08	0.84 ± 0.11
Pituitary gland	5.30 ± 0.66	5.65 ± 1.10	5.24 ± 1.07	5.53 ± 0.79	5.80 ± 0.75
Spleen	0.28 ± 0.02	0.28 ± 0.03	0.28 ± 0.02	0.27 ± 0.02	0.22 ± 0.07
Thymus	0.16 ± 0.01	0.17 ± 0.03	0.16 ± 0.02	0.16 ± 0.01	0.12 ± 0.06
Ovary-R	21.68 ± 1.89	23.06 ± 5.77	23.37 ± 4.16	23.89 ± 4.23	22.11 ± 3.78
Ovary-L	23.21 ± 2.78	22.07 ± 2.56	22.79 ± 1.92	21.39 ± 2.27	22.12 ± 5.20
Uterus	0.21 ± 0.05	0.24 ± 0.06	0.34 ± 0.20	0.26 ± 0.11	0.20 ± 0.08
Salivary gland	0.20 ± 0.02	0.21 ± 0.02	0.21 ± 0.02	0.22 ± 0.01	0.17 ± 0.02 *
Thyroid gland-R (mg)	4.52 ± 1.45	4.20 ± 1.17	4.77 ± 0.52	4.69 ± 1.85	5.00 ± 1.40
Thyroid gland-L (mg)	4.67 ± 0.50	4.01 ± 0.76	4.20 ± 0.82	4.01 ± 1.30	4.49 ± 1.87

R, right; L, left. Data are expressed as means ± SD (*n* = 5–7/group). * Significantly different from the vehicle control group (*p* < 0.05).

**Table 6:** Hematological parameters after the 28-day repeated oral toxicity study.

	Groups (mg/kg/day)				
Parameter	0	12.5	25	50	100
Male					
WBC (×10^3^ cells/μL)	8.1 ± 2.3	6.6 ± 1.8	9.1 ± 2.1	7.4 ± 2.3	8.0 ± 1.5
RBC (×10^6^ cells/μL)	8.2 ± 0.2	8.3 ± 0.4	8.2 ± 0.2	8.0 ± 0.8	8.1 ± 0.3
HGB (g/dL)	16.2 ± 0.5	15.9 ± 0.7	15.9 ± 0.4	15.2 ± 1.4	15.8 ± 0.6
HCT (%)	48.6 ± 1.4	48.7 ± 1.9	48.9 ± 1.6	47.1 ± 5.2	49.3 ± 2.0
MCV (fL)	59.1 ± 1.1	59.0 ± 1.3	59.8 ± 1.3	58.7 ± 2.5	60.7 ± 1.6
MCH (pg)	19.7 ± 0.5	19.2 ± 0.5	19.4 ± 0.6	19.0 ± 0.7	19.5 ± 0.4
MCHC (g/dL)	33.3 ± 0.3	32.6 ± 0.5	32.6 ± 0.5	32.5 ± 0.8 *	32.0 ± 0.4 *
PLT (×10^3^ cells/μL)	1083.8 ± 128.1	993.0 ± 48.8	1001.7 ± 44.8	999.4 ± 84.0	984.0 ± 106.1
NEUT (% of WBC)	11.6 ± 3.5	11.4 ± 2.1	11.6 ± 3.9	15.2 ± 2.5	15.6 ± 5.5
LYM (% of WBC)	84.0 ± 3.8	85.0 ± 2.1	83.4 ± 4.2	80.9 ± 2.8	80.0 ± 5.5
MONO (% of WBC)	2.1 ± 0.8	1.8 ± 0.4	2.4 ± 0.9	2.1 ± 0.6	2.4 ± 0.3
EOS (% of WBC)	1.0 ± 0.2	0.7 ± 0.4	0.7 ± 0.2	0.7 ± 0.3	0.7 ± 0.1
BASO (% of WBC)	0.6 ± 0.3	0.5 ± 0.2	0.7 ± 0.3	0.5 ± 0.2	0.6 ± 0.1
Retic (%)	2.7 ± 0.4	2.5 ± 0.3	2.6 ± 0.3	2.6 ± 0.3	3.0 ± 0.5
Female					
WBC (×10^3^ cells/μL)	5.1 ± 2.1	5.1 ± 1.6	5.3 ± 1.4	4.8 ± 1.2	4.7 ± 0.7
RBC (×10^6^ cells/μL)	6.2 ± 2.8	7.6 ± 0.3	7.8 ± 0.3	7.9 ± 0.2 *	7.4 ± 0.2
HGB (g/dL)	14.7 ± 1.1	14.5 ± 0.5	14.9 ± 0.4	15.2 ± 0.8	14.3 ± 0.5
HCT (%)	36.4 ± 16.6	43.8 ± 1.5	45.1 ± 1.7	45.9 ± 1.9	43.6 ± 1.6
MCV (fL)	57.9 ± 1.0	57.9 ± 1.2	58.1 ± 0.9	57.9 ± 1.5	59.2 ± 1.4
MCH (pg)	18.7 ± 2.2	19.2 ± 0.3	19.2 ± 0.6	19.2 ± 0.6	19.4 ± 0.4
MCHC (g/dL)	32.2 ± 3.3	33.2 ± 0.6	33.0 ± 0.7	33.2 ± 0.7	32.8 ± 0.3
PLT (×10^3^ cells/μL)	1039.8 ± 89.8	1057.3 ± 149.4	1064.6 ± 98.3	1111.7 ± 40.4	1063.7 ± 75.0
NEUT (% of WBC)	12.1 ± 5.2	12.9 ± 4.8	9.5 ± 1.9	9.7 ± 1.8	15.2 ± 2.8
LYM (% of WBC)	83.6 ± 4.4	84.1 ± 5.0	85.4 ± 4.2	85.7 ± 2.5	75.9 ± 12.4
MONO (% of WBC)	2.2 ± 0.8	1.6 ± 0.6	1.8 ± 0.5	2.5 ± 1.0	2.3 ± 1.0
EOS (% of WBC)	0.9 ± 0.5	0.4 ± 0.3 *	0.6 ± 0.2	0.7 ± 0.3	0.5 ± 0.2
BASO (% of WBC)	0.4 ± 0.1	0.4 ± 0.2	0.4 ± 0.1	0.5 ± 0.2	0.3 ± 0.1
Retic (%)	2.0 ± 0.2	2.2 ± 0.5	2.3 ± 0.3	2.1 ± 0.4	1.9 ± 1.0

WBC, white blood cell count; RBC, red blood cell count; HCB, hemoglobin; HCT, hematocrit; MCV, mean corpuscular volume; MCH, mean corpuscular hemoglobin; MCHC, mean corpuscular hemoglobin concentration; PLT, platelet count; NEUT, neutrophil; LYM, lymphocyte; MONO, monocyte; EOS, eosinophil; BASO, basophil; Retic, reticulocyte. Data are expressed as means ± SD (*n* = 5–7/group). * Significantly different from the vehicle control group (*p* < 0.05).

**Table 7:** Clinical biochemistry after the 28-day repeated oral toxicity study.

	Groups (mg/kg/day)				
Parameter	0	12.5	25	50	100
Male					
ALT (U/L)	43.0 ± 8.5	45.0 ± 13.0	47.6 ± 11.3	43.3 ± 9.5	52.3 ± 5.9
AST (U/L)	109.2 ± 17.2	105.3 ± 20.1	98.7 ± 17.6	103.2 ± 21.8	104.4 ± 15.8
ALP (U/L)	177.4 ± 40.4	205.4 ± 18.9	208.4 ± 22.9	215.7 ± 23.9 *	186.6 ± 10.6
GGT (U/L)	2.2 ± 0.8	2.2 ± 1.0	2.5 ± 0.5	2.2 ± 0.4	2.5 ± 0.5
BUN (mg/dL)	15.5 ± 4.2	12.7 ± 3.0	12.8 ± 2.4	13.5 ± 4.2	15.0 ± 2.9
CREA (mg/dL)	0.7 ± 0.0	0.7 ± 0.0	0.7 ± 0.0	0.8 ± 0.1	0.7 ± 0.1
TP (g/L)	5.9 ± 0.3	5.9 ± 0.3	6.0 ± 0.3	6.1 ± 0.5	6.2 ± 0.4
ALB (g/dL)	3.4 ± 0.1	3.4 ± 0.1	3.4 ± 0.1	3.4 ± 0.1	3.4 ± 0.1
T-CHO (mg/dL)	87.0 ± 10.7	93.3 ± 20.0	105.3 ± 12.1	104.0 ± 16.8	117.1 ± 16.5 *
GLU (mg/dL)	132.0 ± 19.5	120.4 ± 17.6	118.6 ± 20.1	132.8 ± 15.5	78.7 ± 14.4 *
TG (mmol/L)	64.4 ± 18.0	64.9 ± 23.2	68.3 ± 22.9	65.3 ± 24.8	64.3 ± 20.7
T-BIL (mg/dL)	0.2 ± 0.1	0.2 ± 0.0	0.2 ± 0.0	0.2 ± 0.1	0.2 ± 0.0
D-BIL (mg/dL)	0.0 ± 0.0	0.0 ± 0.0	0.0 ± 0.0	0.0 ± 0.0	0.0 ± 0.0
LDH (U/L)	968.2 ± 207.1	861.3 ± 387.3	645.9 ± 348.8	989.0 ± 262.4	912.0 ± 273.3
CK (U/L)	454.0 ± 107.7	460.9 ± 207.0	374.1 ± 192.4	446.5 ± 243.0	390.3 ± 128.4
UA (mg/dL)	1.8 ± 0.2	1.6 ± 0.4	1.4 ± 0.4	1.6 ± 0.4	1.4 ± 0.2
CA (mg/dL)	9.5 ± 0.2	10.7 ± 1.5	10.0 ± 0.8	10.3 ± 1.2	11.0 ± 2.5
IP (mg/dL)	8.9 ± 0.8	9.2 ± 0.9	8.4 ± 0.9	9.5 ± 1.5	9.8 ± 1.1
HDL (mg/dL)	77.4 ± 7.9	78.7 ± 14.0	85.4 ± 7.9	85.3 ± 10.4	93.4 ± 10.5
LDL (mg/dL)	19.8 ± 5.0	20.9 ± 6.6	26.9 ± 3.8	26.5 ± 5.3	27.6 ± 5.3
Female					
ALT (U/L)	39.6 ± 6.5	34.7 ± 9.9	32.7 ± 5.6	33.0 ± 6.5	41.5 ± 5.9
AST (U/L)	107.0 ± 9.9	103.3 ± 13.6	101.3 ± 18.2	92.9 ± 17.0	83.7 ± 17.2
ALP (U/L)	148.8 ± 32.9	147.4 ± 27.1	142.9 ± 53.0	107.4 ± 20.3	116.5 ± 21.0
GGT (U/L)	3.0 ± 0.7	2.4 ± 0.5	2.9 ± 0.4	3.0 ± 0.0	2.6 ± 0.5
BUN (mg/dL)	11.8 ± 1.2	13.0 ± 3.2	12.8 ± 2.9	16.4 ± 1.6 *	14.8 ± 4.1
CREA (mg/dL)	0.6 ± 0.1	0.7 ± 0.1	0.7 ± 0.1	0.7 ± 0.1	0.9 ± 0.6
TP (g/L)	5.7 ± 0.2	6.0 ± 0.4	6.1 ± 0.3	6.2 ± 0.3	6.8 ± 0.6 *
ALB (g/dL)	3.4 ± 0.1	3.5 ± 0.2	3.5 ± 0.1	3.5 ± 0.1	3.6 ± 0.1
T-CHO (mg/dL)	79.4 ± 11.6	92.1 ± 21.5	86.6 ± 13.3	101.0 ± 22.7	163.2 ± 53.3 *
GLU (mg/dL)	110.6 ± 26.9	109.1 ± 15.1	118.9 ± 14.1	112.0 ± 10.0	86.0 ± 34.3
TG (mmol/L)	29.4 ± 9.0	33.6 ± 6.6	33.0 ± 11.8	35.9 ± 14.0	49.6 ± 27.8
T-BIL (mg/dL)	0.2 ± 0.0	0.2 ± 0.0	0.2 ± 0.0	0.2 ± 0.0	0.2 ± 0.0
D-BIL (mg/dL)	0.1 ± 0.0	0.1 ± 0.0	0.1 ± 0.0	0.1 ± 0.0	0.1 ± 0.1
LDH (U/L)	1492.0 ± 481.2	1488.1 ± 503.5	1379.7 ± 604.7	1598.4 ± 628.0	1403.6 ± 830.7
CK (U/L)	375.0 ± 64.4	397.3 ± 61.3	394.6 ± 131.9	357.0 ± 119.1	326.7 ± 85.4
UA (mg/dL)	1.4 ± 0.2	1.6 ± 0.3	1.4 ± 0.3	1.4 ± 0.2	1.1 ± 0.3
CA (mg/dL)	9.0 ± 0.2	9.7 ± 1.2	9.7 ± 1.1	9.7 ± 1.2	10.7 ± 1.6
IP (mg/dL)	7.3 ± 1.0	7.5 ± 0.5	7.6 ± 0.9	8.3 ± 1.1	8.9 ± 3.1
HDL (mg/dL)	71.6 ± 7.7	78.9 ± 16.5	76.0 ± 10.0	88.1 ± 15.8	136.2 ± 29.8 *
LDL (mg/dL)	13.2 ± 4.4	16.6 ± 3.8	16.3 ± 3.7	16.4 ± 6.9	31.8 ± 12.6 *

ALT, alanine aminotransferase; AST, aspartate aminotransferase; ALP, alkaline phosphatase; GGT, gamma-glutamyl transferase; BUN, blood urea nitrogen; CREA, creatinine; TP, total protein; ALB, albumin; T-CHOL, cholesterol; GLU, glucose; TG, triglyceride; T-BIL, total bilirubin; D-BIL, direct bilirubin; LDH, lactate dehydrogenase; CK, creatine kinase; UA, uric acid; CA, calcium; IP, phosphorus; HDL, high-density lipoprotein; LDL, low-density lipoprotein. Data are expressed as means ± SD (*n* = 5–7/group). * Significantly different from the vehicle control group (*p* < 0.05).

## Data Availability

The original contributions presented in the study are included in the article/material, further inquiries can be directed to the corresponding authors.
